# Lung versus gut exposure to air pollution particles differentially affect metabolic health in mice

**DOI:** 10.1186/s12989-023-00518-w

**Published:** 2023-03-09

**Authors:** Angela J. T. Bosch, Theresa V. Rohm, Shefaa AlAsfoor, Andy J. Y. Low, Lena Keller, Zora Baumann, Neena Parayil, Marc Stawiski, Leila Rachid, Thomas Dervos, Sandra Mitrovic, Daniel T. Meier, Claudia Cavelti-Weder

**Affiliations:** 1 Department of Biomedicine, University of Basel, 4031 Basel, Switzerland; 2 Department of Laboratory Medicine, University Hospital Basel, 4031 Basel, Switzerland; 3 Clinic of Endocrinology, Diabetes and Metabolism, University Hospital Basel, 4031 Basel, Switzerland; 4 Department of Endocrinology, Diabetology and Clinical Nutrition, University Hospital Zurich (USZ) and University of Zurich (UZH), Rämistrasse 100, 8009 Zurich, Switzerland

**Keywords:** Air pollution, Diesel exhaust particles, Particulate matter, Exposure route, Gut exposure, Lung exposure, Glucose metabolism, Metabolic disease, Insulin secretion

## Abstract

**Background:**

Air pollution has emerged as an unexpected risk factor for diabetes. However, the mechanism behind remains ill-defined. So far, the lung has been considered as the main target organ of air pollution. In contrast, the gut has received little scientific attention. Since air pollution particles can reach the gut after mucociliary clearance from the lungs and through contaminated food, our aim was to assess whether exposure deposition of air pollution particles in the lung or the gut drive metabolic dysfunction in mice.

**Methods:**

To study the effects of gut versus lung exposure, we exposed mice on standard diet to diesel exhaust particles (DEP; NIST 1650b), particulate matter (PM; NIST 1649b) or phosphate-buffered saline by either intratracheal instillation (30 µg 2 days/week) or gavage (12 µg 5 days/week) over at least 3 months (total dose of 60 µg/week for both administration routes, equivalent to a daily inhalation exposure in humans of 160 µg/m^3^ PM_2.5_) and monitored metabolic parameters and tissue changes. Additionally, we tested the impact of the exposure route in a “prestressed” condition (high-fat diet (HFD) and streptozotocin (STZ)).

**Results:**

Mice on standard diet exposed to particulate air pollutants by intratracheal instillation developed lung inflammation. While both lung and gut exposure resulted in increased liver lipids, glucose intolerance and impaired insulin secretion was only observed in mice exposed to particles by gavage. Gavage with DEP created an inflammatory milieu in the gut as shown by up-regulated gene expression of pro-inflammatory cytokines and monocyte/macrophage markers. In contrast, liver and adipose inflammation markers were not increased. Beta-cell secretory capacity was impaired on a functional level, most likely induced by the inflammatory milieu in the gut, and not due to beta-cell loss. The differential metabolic effects of lung and gut exposures were confirmed in a “prestressed” HFD/STZ model.

**Conclusions:**

We conclude that separate lung and gut exposures to air pollution particles lead to distinct metabolic outcomes in mice. Both exposure routes elevate liver lipids, while gut exposure to particulate air pollutants specifically impairs beta-cell secretory capacity, potentially instigated by an inflammatory milieu in the gut.

**Supplementary Information:**

The online version contains supplementary material available at 10.1186/s12989-023-00518-w.

## Background

The World Health Organization has identified air pollution as one of the ten leading threats to global health [1]. Depending on the model used, ambient air pollution has been estimated to contribute to 3.3–8.7 million premature deaths annually, which is almost up to one-fifth of all deaths globally [2–5]. Mortality due to air pollution is projected to double by 2050 as urbanization and air pollution are steadily increasing [4]. The best-studied health effects caused by particle exposure are respiratory diseases and cardiovascular complications. However, air pollution has also emerged as an unexpected risk factor for diabetes in many epidemiological [6–11] and rodent studies [12, 13]. This association even occurs at air pollution levels below those designated as safe by the World Health Organization [14], suggesting that a further reduction in particle concentrations would have significant positive health impacts.

To date, the lung has been considered as the main target organ of air pollution. So far, it is believed that air pollution induces lung inflammation and subsequently leads to systemic inflammation, insulin resistance and a diabetic phenotype. Alternatively, air pollution-induced diabetes could be mediated via gut exposure. Both in mice and humans, inhaled particles are known to be cleared via mucociliary escalator from the upper airways toward the larynx and followed by passage through the gastrointestinal tract with subsequent fecal excretion [15–17]. Pollutants contaminating food and water account for additional sources of gut exposure [18]. It has been estimated that a person on a typical western diet ingests up to 10^12^–10^14^ particles daily only from food additives [19]. Underlining the clinical relevance of gut exposure, air pollution has been linked to increased incidence of many gastrointestinal tract diseases, such as inflammatory bowel disease, cancer of the gastrointestinal tract, appendicitis and irritable bowel syndrome [20–22]. Moreover, air pollutants are known to alter gut microbiota [23–25], increase gut leakiness [26], and induce gut inflammation [27], all factors known to be interlinked with diabetes.

So far, the development of diabetes upon air pollution exposure has been attributed to an insulin resistance phenotype [28–31]. Also, hepatic steatosis and increased plasma lipids, both typical features of metabolic disease, have been reported in mice exposed to air pollutants [32, 33]. Despite the robust data linking air pollution and features of metabolic disease, it is unknown whether lung and gut exposure to air pollutants lead to differential health effects. Most studies performed in mice use inhalation chambers. Thereby, differential health effects of gut versus lung exposure cannot be addressed as mucociliary clearance as well as ingestion of particle residues on food, the bedding or fur of the mice might contribute to oral exposure. Our aim was to assess the role of the exposure route in the pathogenesis of metabolic disease, especially diabetes. Therefore, we separately exposed mice via lung or gut to pollutants with as little contamination of the other route as possible and studied the differential metabolic health effects. The standard diet model allowed us to assess the exposure route-induced development of metabolic disease with as little confounding factors as possible, while the HFD/ STZ model served as a “prestressed” condition to evaluate disease progression upon the differential exposure routes. A better understanding on the relationship between the exposure route and outcomes such as diabetes is crucial to better understand the disease mechanism and eventually develop targeted prevention and treatment strategies.

## Results

### Separate lung exposure to air pollution particles does not induce diabetes in mice fed a standard diet

**Fig. 1:**
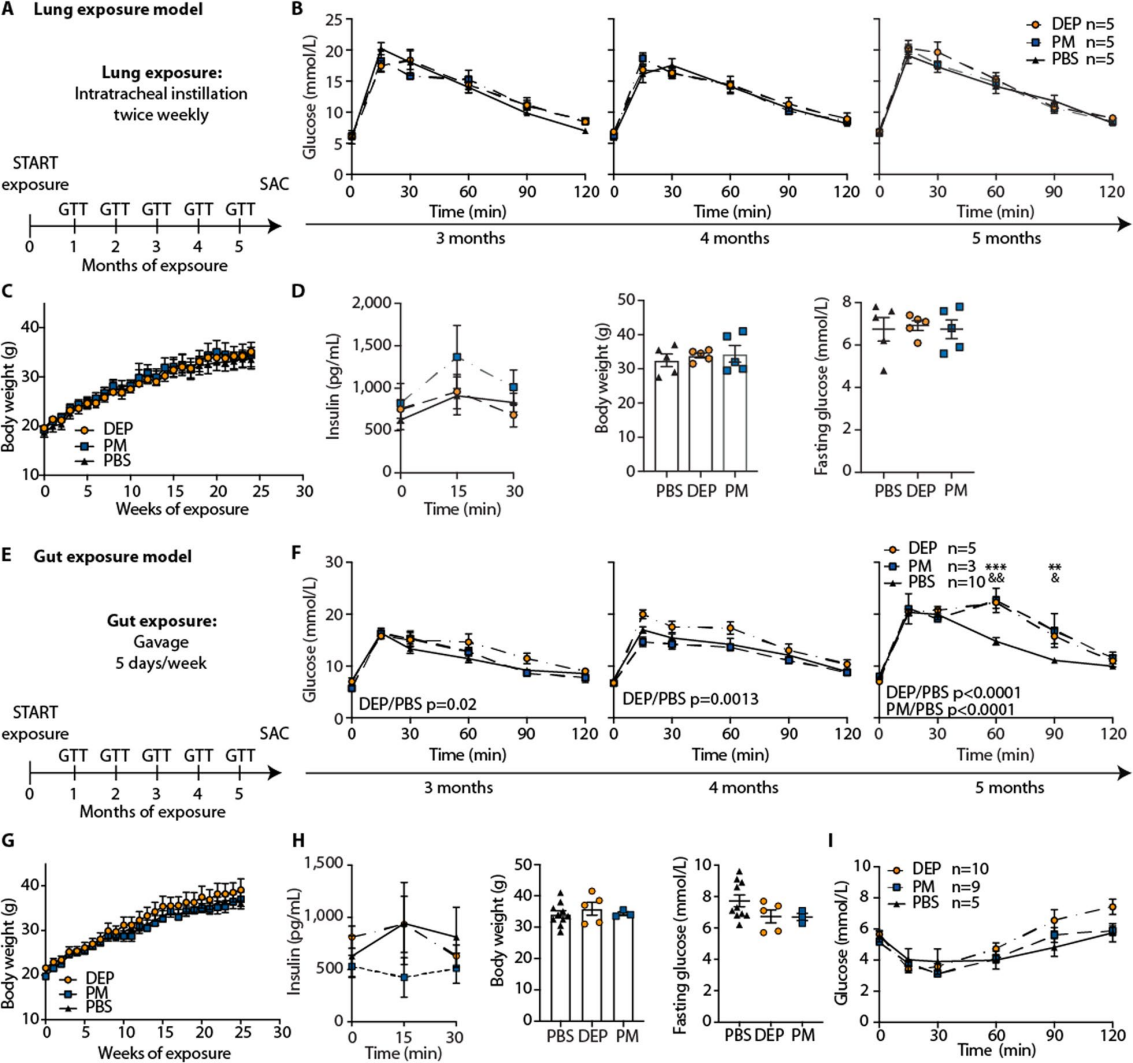
Separate lung exposure to air pollution particles does not induce diabetes, while gut exposure induces glucose intolerance and impaired insulin secretion in mice fed a standard diet. **A** Schematic illustration of lung exposure model. Wild-type mice were intratracheally instilled with 30 µg diesel exhaust particles (DEP), particulate matter (PM) or PBS twice weekly starting at 5–6 weeks of age for 6 months. **B** Time course of glucose tolerance tests (GTT) for months 3–5. **C** Body weight over time. **D** Insulin, body weight and fasting glucose after 5 months of exposure. **E** Schematic illustration of gut exposure model. Wild-type mice were treated with 12 µg diesel exhaust particles (DEP), particulate matter (PM) or PBS 5 times per week via gavage starting at 5–6 weeks of age for up to 6 months. **F** Time course of glucose tolerance tests (GTT) for months 3–5. **G** Body weight over time.** H** Insulin, body weight and fasting glucose after 4 months of gavage. **I** ITT after 3 months of treatment. Data are presented as mean ± SEM of 5 mice per group from one representative experiment. GTT and insulin values were compared by two-way ANOVA, body weight and fasting glucose by a two-tailed, unpaired Mann–Whitney U test (*p < 0.05, **p < 0.01). * indicates significances between 12 µg DEP and PBS controls and & between 12 µg PM and PBS controls

First, we aimed to assess the effect of separate lung exposure on glucose metabolism with as little contamination of air pollution particles to the gut as possible. To this end, we exposed mice on standard diet to diesel exhaust particles (DEP), particulate matter (PM), or PBS (phosphate-buffered saline; control) by deep intratracheal instillation in an upright position (twice weekly 30 µg, Fig. 1A). Mice intratracheally exposed to DEP or PM up to five months did not develop impaired glucose tolerance as assessed by monthly performed glucose tolerance tests (Fig. 1B). There were no changes in insulin, body weight, and fasting glycemia (Fig. 1C, D). Hence, separate lung exposure did not induce a diabetic phenotype upon particulate air pollution exposure in mice on standard diet.

### Separate gut exposure to air pollution particles induces glucose intolerance and impaired insulin secretion in mice fed a standard diet

Next, we assessed the role of separate gut exposure in mediating air pollution-induced diabetes and exposed mice on standard diet to DEP, PM, or PBS by gavage (12 µg 5 days/week, Fig. 1E). Experiments with gavage and intratracheal instillations contained the same weekly dose of 60 µg, equivalent to an inhalational exposure of approximately 160 μg/m^3^, and were carried out simultaneously until the appearance of a diabetic phenotype in one of the groups. Mice exposed to air pollution particles via gavage exhibited glucose intolerance and impaired insulin secretion from approximately 3–4 months onwards (Fig. 1F–H). In contrast, their body weight, fasting glucose, and insulin sensitivity as assessed by ITT (insulin tolerance test) remained unchanged (Fig. 1G–I). Exposure to DEP induced slightly more pronounced effects on glycemia than did PM (Fig. 1F). However, when we compared two different doses of DEP exposure (12 µg and 60 µg 5 days/week), we did not find a dose-dependent impairment of glucose intolerance (Additional file 1: Figure S1). Thus, gut exposure to particles in mice on standard diet led to glucose intolerance as a consequence of impaired insulin secretion, rather than insulin resistance.

### In a “prestressed” condition, gut exposure to air pollution particles also induces glucose intolerance and impaired insulin secretion, while lung exposure does not

**Fig. 2:**
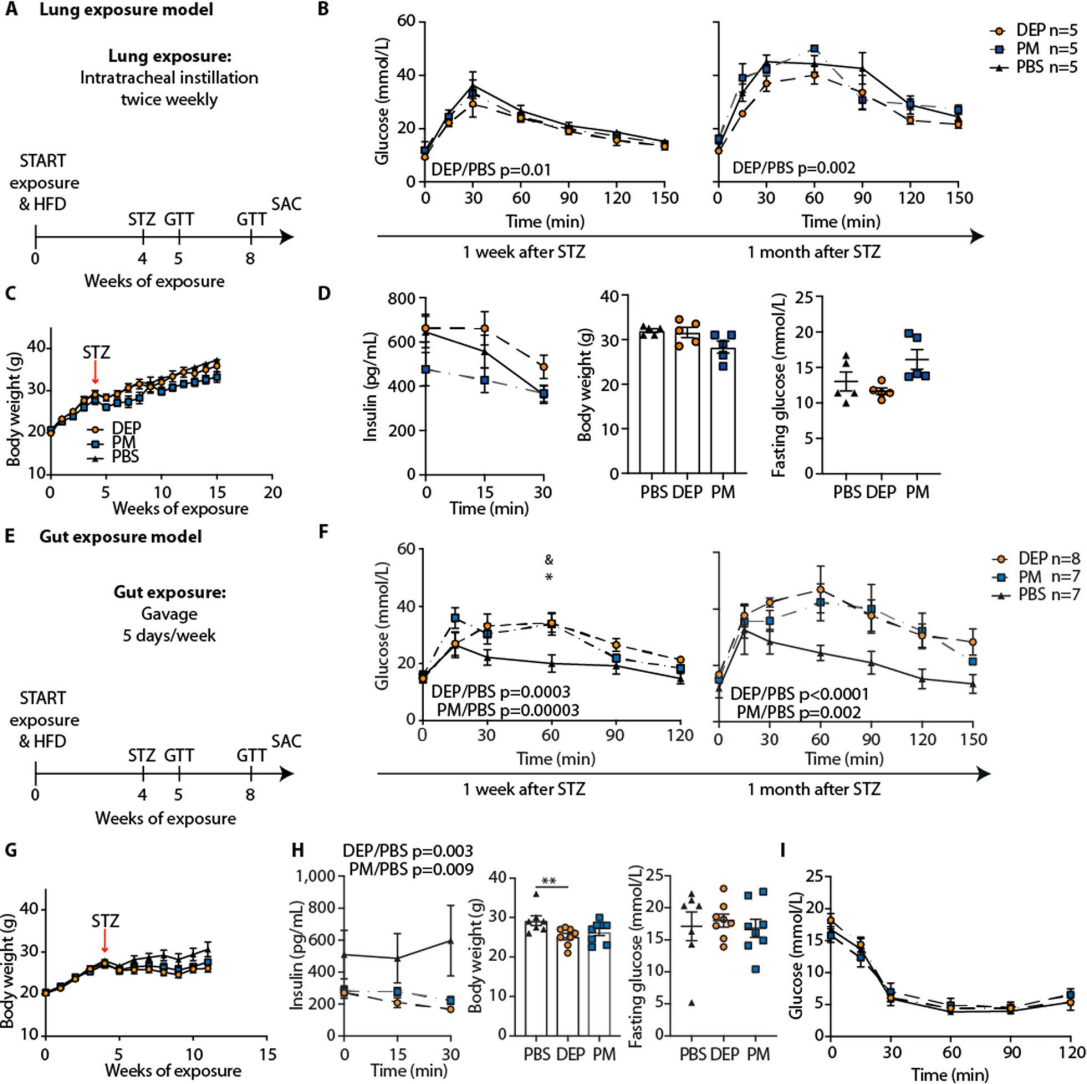
In a “prestressed” condition, gut exposure to air pollution particles also induces glucose intolerance and impaired insulin secretion, while lung exposure does not. Mice on high-fat diet (HFD) were concomitantly treated by intratracheal instillation with DEP, PM or PBS. After 4 weeks of treatment, mice were rendered diabetic by a single dose of streptozotocin (STZ, 120 mg/kg). **A** Schematic illustration of lung exposure model. **B** GTT in HFD/STZ treated mice 1 week and 1 month after STZ injection. **C** Body weight over time. **D** Insulin, body weight and fasting glucose 1 month after STZ injection. **E** Schematic illustration of gut exposure model. **F** GTT in HFD/STZ treated mice 1 week and 1 month after STZ injection. **G** Body weight over time. **H** Insulin, body weight and fasting glucose 1 month after STZ treatment. **I** Insulin tolerance test (ITT) one month after STZ injection. Data are presented as mean ± SEM of 5–8 mice per group from one representative experiment each. GTT and insulin values were compared by two-way ANOVA, body weight and fasting glucose by a two-tailed, unpaired Mann–Whitney U test *p < 0.05, **p < 0.01). * indicates significances between 12 µg DEP and PBS controls and & between 12 µg PM and PBS controls

To corroborate these findings in a “prestressed” condition, we used mice fed a high-fat diet (HFD) treated with a single dose of streptozotocin (STZ). This model combines two key features of type 2 diabetes, namely insulin resistance (upon HFD) and partially reduced beta-cell mass (induced by STZ) to limit beta-cell compensation [34, 35]. Intratracheal instillations with DEP, PM, or PBS were carried out one month before and one month after STZ injection (Fig. 2A). As with standard diet, glucose tolerance did not differ from those of the control mice (Fig. 2B). Also, insulin, body weight and fasting glucose were comparable (Fig. 2C, D). In contrast, HFD/STZ treated mice exposed to air pollution particles via gavage (20 µg 5 days/week, Fig. 2E) developed worsened glucose tolerance and impaired insulin secretion from 5 weeks of exposure onwards (Fig. 2F, H). In addition, the body weights of DEP exposed mice were reduced, reflecting the loss of insulin's anabolic action (Fig. 2G, H). Fasting glucose and insulin sensitivity were unchanged (Fig. 2H, I). Thus, gut exposure to air pollution particles induced glucose intolerance and impaired insulin secretion also in a “prestressed” condition involving HFD/STZ, while lung exposure did not.

### Separate lung exposure to air pollution particles leads to lung inflammation, hypercholesterinemia and increased liver lipids in mice fed a standard diet

**Fig. 3:**
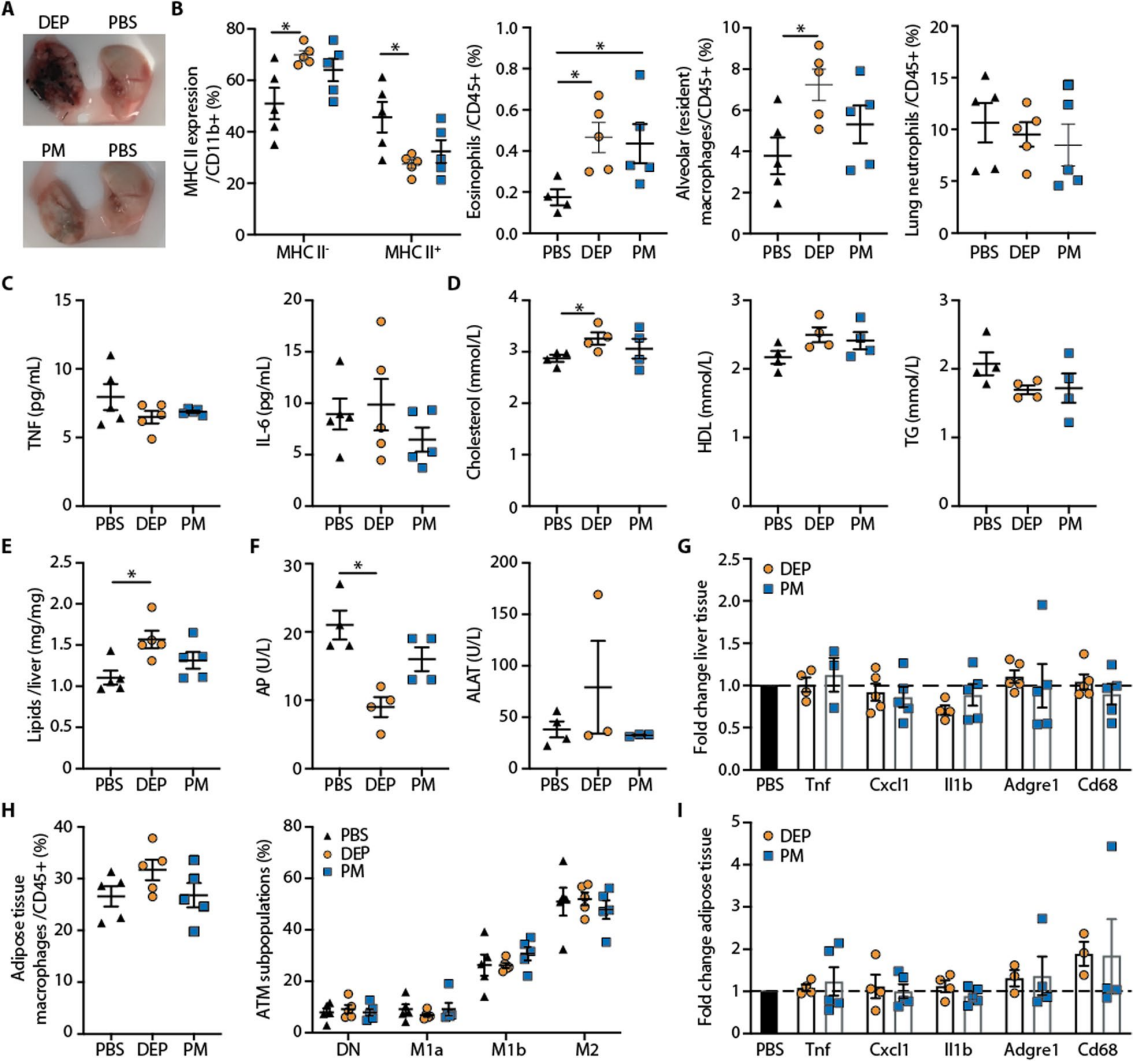
Separate lung exposure to air pollution particles leads to lung inflammation, hypercholesterinemia and increased liver lipids in mice fed a standard diet. **A** Representative picture of lungs from mice intratracheally instilled with diesel exhaust particles (DEP), particulate matter (PM) or PBS. **B** Frequencies of lung monocytes and macrophages among CD11b^+^ cells (MHC II^−^ cells correspond to monocytes and macrophages; MHC II^+^ cells to CD11b^+^ DCs and resident interstitial macrophages). Eosinophils (SiglecF^+^CD11c^+^), alveolar macrophages (SiglecF^+^CD11b^−^) and lung neutrophils (Ly6G^+^CD11b^+^) were gated on CD45^+^ cells (gating strategy Additional file 1: Fig. S2A). **C** Plasma TNF and IL-6. **D** Cholesterol, high-density lipoproteins (HDL), and triglycerides (TG). **E** Liver lipids. **F** Liver enzymes (alkaline phosphatase (AP), alanine transaminase (ALAT)). **G** Inflammatory gene expression of liver normalized to PBS. **H** Frequencies of adipose tissue macrophages (ATM), and their subpopulations defined by CD11c and CD206 and gene expression (gating Additional file 1: Fig. S2B). **I** Inflammatory gene expression in adipose tissue, normalized to PBS. Data are presented as mean ± SEM of 5 mice per group from one experiment compared by a two-tailed, unpaired Mann–Whitney U test (*p < 0.05)

To understand how lung versus gut exposures differentially affect tissue homeostasis, we phenotypically characterized different tissues and analyzed blood parameters in mice fed a standard diet. In mice exposed to air pollution particles via intratracheal instillations, the lungs appeared black, confirming that pollution particles reached the lungs (Fig. 3A). These deposits caused lung inflammation, as evidenced by increased frequencies of eosinophils and monocyte-derived CD11b^+^MHCII^−^ macrophages, while the MHCII^+^ populations consisting of tissue-resident interstitial macrophages and conventional dendritic cells (DCs) were reduced (Fig. 3B; Additional file 1: Figures S2,3). In addition, the frequencies of tissue-resident alveolar macrophages were significantly increased upon DEP but not PM exposure, while neutrophils remained unchanged upon chronic lung exposure (Fig. 3B; Additional file 1: Figure S2).

Lung exposure did not increase inflammatory markers TNF and IL-6 systemically (Fig. 3C). Total cholesterol was elevated in DEP-exposed mice, while HDL and triglycerides were unchanged (Fig. 3D). Hypercholesterinemia was mirrored by increased liver lipids in mice intratracheally exposed to DEP (assay for unsaturated organic compounds; Fig. 3E). Except for a decrease in Alkaline Phosphatase (AP) in DEP-exposed mice, liver enzymes and inflammatory gene expression were unaffected by lung exposure (Fig. 3F, G). Adipose tissue showed no changes in macrophage abundance including subpopulations and inflammatory gene expression (Fig. 3H, I). Thus, lung exposure to air pollution particles caused lung inflammation as shown by elevated eosinophils and monocyte-derived macrophages, together with increased systemic cholesterol levels and liver lipids in DEP-exposed mice.

### Separate gut exposure to air pollution particles increases liver lipids and induces gut inflammation in mice fed a standard diet

**Fig. 4:**
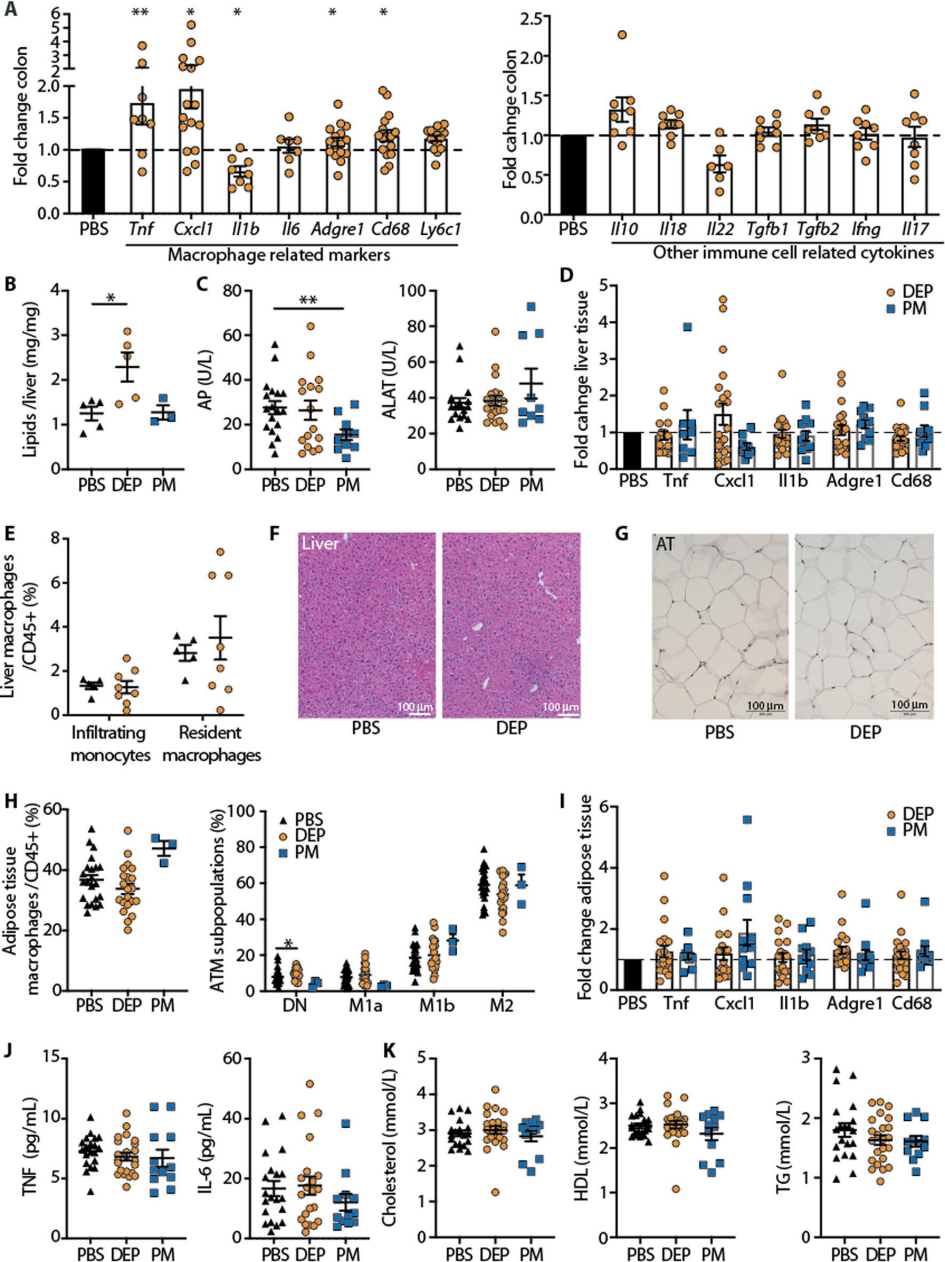
Separate gut exposure to air pollution particles increases liver lipids and induces gut inflammation in mice fed a standard diet. Wild-type mice were treated with 12 µg diesel exhaust particles (DEP), particulate matter (PM) or PBS 5 times per week via gavage for up to 6 months. **A** Gene expression of immune cell markers in the colon. **B** Liver lipids. **C** Liver enzymes alkaline phosphatase (AP) and alanine transaminase (ALAT). **D** Inflammatory gene expression in liver tissue relative to PBS treated controls. **E** Liver macrophages in mice exposed to DEP or PBS via gavage. **F** H&E staining of liver tissue. **G** H&E staining of adipose tissue. **H** Frequencies of adipose tissue macrophages (ATM; F4/80^+^CD11b^+^ among CD45^+^), and their subpopulations defined by CD11c and CD206. **I** Inflammatory gene expression in adipose tissue relative to PBS treated controls. **J** Plasma TNF and IL-6. **K** Cholesterol, high-density lipoproteins (HDL), and triglycerides (TG). Pooled data of 2–3 independent experiments, with each data point representing an individual mouse. *p < 0.05, **p < 0.01, ***p < 0.001, unpaired Mann–Whitney U test with two tailed distribution

Next, we analyzed different tissues and the blood of mice fed standard diet and exposed to particulate air pollutants via gut exposure. We found up-regulated gene expression of pro-inflammatory cytokines (i.e., *Tnf, Cxcl1*) and monocyte/macrophage markers (i.e., *Cd68, Ly6c1*, Fig. 4A) in the gut, consistent with an innate immune response. Expression markers of other immune cells such as of the adaptive immune system, however, were not increased.

Similar to the lung exposure group, gut exposure elevated liver lipids in DEP-exposed mice, while AP levels were reduced in the PM-group (Fig. 4B, C). Inflammatory gene expression and myeloid cells in the liver were unaltered upon gut exposure (Fig. 4D, E). In addition, the livers appeared histologically normal (Fig. 4F). The absence of liver inflammation was further confirmed by hepatic expression of acute phase genes, which were not elevated upon exposure to air pollution (Additional file 1: Figure S4).

Adipose tissue also had no signs of inflammation as shown by normal morphology, unchanged macrophages and their subpopulations as well as unaltered inflammatory gene expression (Fig. 4G–I). Also systemically in the blood, we did not find elevated TNF and IL-6 levels (Fig. 4J) or lipids upon gut exposure as shown by total cholesterol, HDL and triglyceride levels (Fig. 4K). Hence, gut exposure to DEP led to an innate immune response in the gut and increased liver lipids, while liver and adipose inflammation were absent and systemic TNF and IL-6 not increased.

### Impaired insulin secretion upon orally administered air pollution particles in mice fed a standard diet is due to a functional beta-cell defect, but not a reduction in beta-cell mass

**Fig. 5:**
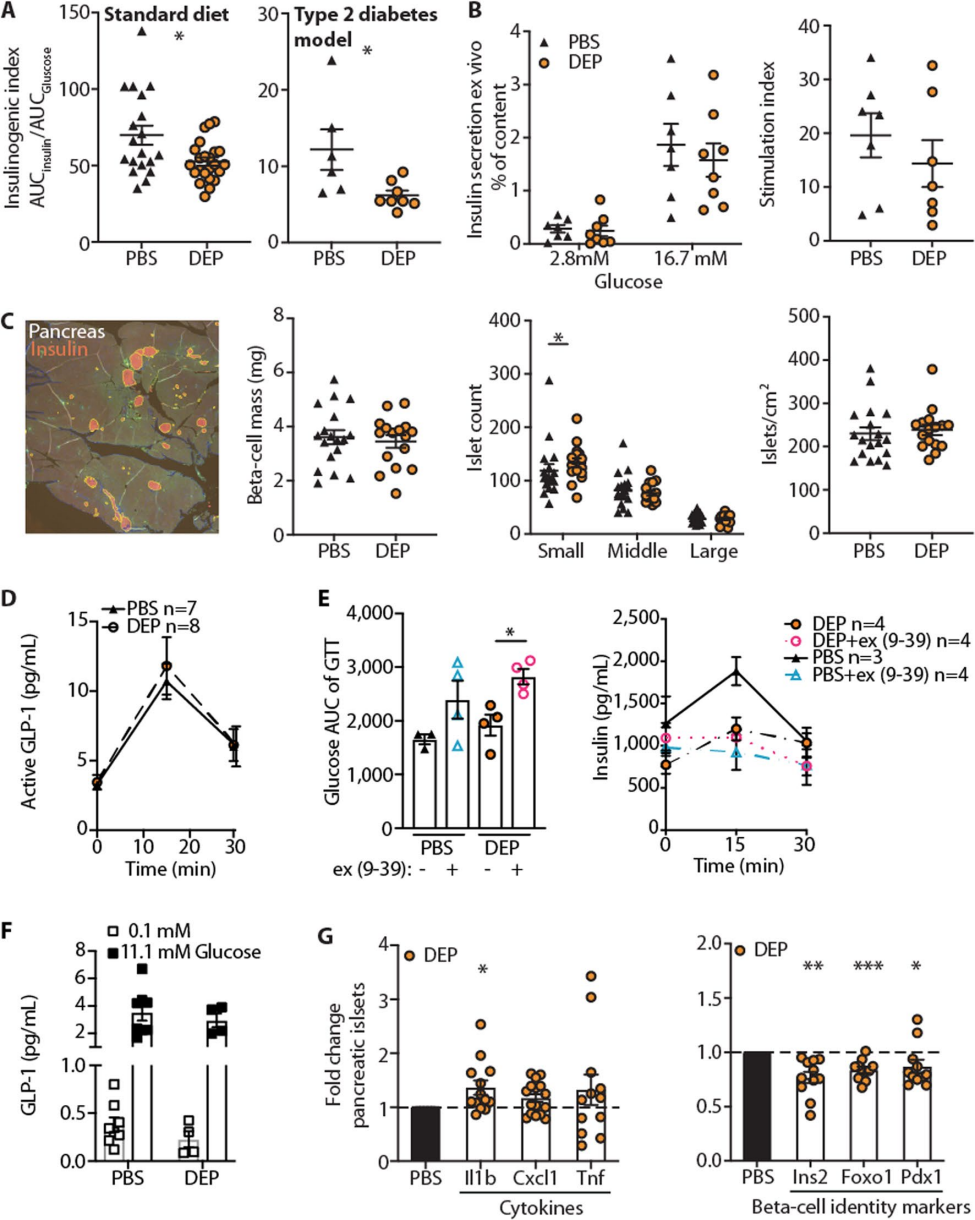
Impaired insulin secretion upon orally administered air pollution particles in mice fed a standard diet is due to a functional beta-cell defect, but not a reduction in beta-cell mass. Wild-type mice were exposed to diesel exhaust particles (DEP) or PBS for 6 months via gavage. **A** Insulinogenic index (defined as the ratio of the areas under the curve of insulin and glucose) in wild type mice on standard diet and HFD/STZ treated wild type mice. **B** Ex vivo glucose-stimulated insulin secretion of isolated islets from mice exposed to DEP or PBS via gavage and stimulation index. Insulin is shown as % of content. **C** Representative insulin staining in pancreatic tissue for analysis of beta-cell mass, number of islets, and size distribution (small 10–1000 µm^2^, middle 1000–10,000 µm^2^, large 10,000–100,000 µm^2^), two data points per mouse. **D** Active GLP-1 in oral GTT. **E** Glucose area under the curve (AUC) of oral GTT and insulin after GLP-1 antagonist exendin (9–39) injection. **F** GLP-1 secretion of primary colon cultures treated ex vivo with 125 µg/mL DEP or PBS. **G** Gene expression in islets from exposed mice relative to controls. Data are presented as mean ± SEM, pooled data from 2 to 4 independent experiments, with each data point representing an individual mouse, except panel (**C**–**E**). **D**, **E** Data from one experiment, with each data point representing an individual mouse. *p < 0.05, **p < 0.01, ***p < 0.001, unpaired Mann–Whitney U test with two tailed distribution

Since our predominant metabolic phenotype was glucose intolerance due to impaired insulin secretion rather than insulin resistance, we further investigated the role of beta-cell dysfunction upon gavage with particulate air pollutants. As shown during the glucose tolerance tests, mice exposed to particulate air pollutants via gavage were unable to mount a compensatory increase in insulin to counter-regulate hyperglycemia (Fig. 1F). Impaired beta-cell function was confirmed by a reduced insulinogenic index (ratio of the area under the curve for insulin and glucose) in both the standard diet and the “prestressed” condition (HFD/STZ) upon gavage with particulate air pollutants (Fig. 5A). Ex vivo glucose-stimulated insulin secretion, however, was comparable between mice exposed to particles via gavage and control mice (Fig. 5B), suggesting that impaired beta-cell function depended on intact cell-to-cell communication. To discriminate whether impaired insulin secretion was due to a functional beta-cell defect or reduced beta-cell mass, we quantified beta-cell mass upon oral exposure to air pollution particles or PBS. We found unchanged beta-cell mass and number of islets except for the small islets, which were numerically increased in DEP exposed mice (Fig. 5C). This finding supported the notion of a functional beta-cell defect rather than morphologically reduced beta-cell mass (i.e. due to apoptosis).

As air pollution particles applied by gavage could interact with enteroendocrine cells in the gut wall and alter the secretion of insulin-secretory hormones such as glucagon-like peptide-1 (GLP-1), we performed an oral glucose tolerance test to assess GLP-1 release. GLP-1 secretion was not impaired upon oral glucose stimulation (Fig. 5D). Accordingly, glucose tolerance and insulin levels were comparable after blocking GLP-1 by its receptor antagonist exendin (9–39) in DEP exposed mice compared to controls (Fig. 5E). Furthermore, primary colon cultures treated with DEP or PBS ex vivo showed similar GLP-1 secretion (Fig. 5F).

As we had found gut inflammation in mice exposed to DEP via gavage, inflammatory processes could be involved in mediating air pollution-induced beta-cell dysfunction. We found an increase in the pro-inflammatory cytokine *Il1b* in islets from DEP exposed mice, while gene expression of *Cxcl1* and *TNF* was comparable (Fig. 5G). Islet inflammation was accompanied by beta-cell dedifferentiation as shown by reduced expression of the beta-cell identity genes *Ins2*, *Foxo1*, and *Pdx1* (Fig. 5G), a finding that has previously also been linked to islet inflammation [36]. Conjunctly, these data indicate that beta-cell dysfunction contributed to the development of air pollution-induced diabetes, potentially instigated by an inflammatory milieu in the gut spreading to the islets of Langerhans.

## Discussion

Our study shows that lung and gut exposure to particulate air pollutants have distinct metabolic effects. The administration mode of separate lung and gut exposures to particulate air pollutants with as little contamination by the other route as possible was designed to specifically assess exposure-dependent metabolic effects. Tracer studies showed that intratracheal instillation leads to similar retention times in the lower respiratory tract as inhalation [37], rendering it a suitable model for lung exposure. It has been estimated that about 50% of inhaled particles undergo long-term retention with 10% of initially deposited particles retained in the lungs after 9 months [15]. Although we cannot exclude that small quantities of particulate air pollutants administered by deep intratracheal instillations reached the gastrointestinal tract, the risk for contamination was minimized by an upright positioning of the mice after instillation [38]. Even in the case of spillover of particles into the gut after intratracheal instillation, we assume that the number of particles reaching the gastrointestinal tract would be substantially lower than in the mice orally exposed by gavage. This is supported by the fact that we observed clear differences in the diabetic phenotype between mice exposed via lung or gut exposure. A limitation of separate lung and gut exposure is that synergistic effects between the two exposure routes cannot be taken into account and that intratracheal instillations require anesthesia unlike gavage, which might by itself result in changes in glucose homeostasis. However, glucose tolerance was comparable in PBS treated control mice of the gut and lung exposure groups, thereby making it unlikely that anesthesia introduced a bias.

We chose a similar cumulative weekly dose as previously used in other studies in the field [12, 39], corresponding to an inhalation exposure of approximately 160 μg/m^3^. This exposure is about 13.5times higher than the annual standard of 12 μg/m^3^ PM_2.5_ in the United States and corresponds to highly polluted areas such as Delhi during the winter months [40]. Interestingly, we did not find a dose dependency with higher concentrations. This might be explained by a non-linear relationship, or by the fact that we already reached the maximum response with the lower dose. As a proof-of-principle we chose equivalent doses for lung and gut exposures to test whether exposure deposition drives metabolic dysfunction. Similarly, equal doses via gut, lung or intravenous exposure routes were used to assess the deposition effect on microvascular function [41]. However, it is challenging to accurately estimate the exact dose of air pollution particles that each organ has to “deal with” in a real-life setting. Substantial amounts (about 50%) of inhaled particles reach the gut already within only three hours [15]. Mucociliary clearance with subsequent gut exposure is the removal mechanism primarily for larger particles, while smaller particles can gain access to the periphery of the lungs [15]. Compared to gut exposure, particles reaching the lung could be trapped in the alveoli and thereby exert longer-lasting effects. Hence, our gut exposure model (by gavage) represents mainly the effects of lager particles that pass through the gut, while lung exposure (by intratracheal instillation) represents the effects of smaller particles reaching the periphery of the lungs.

Besides the administration mode and the dose of the pollutants, it is important to determine which chemical compound(s) could be responsible for the observed health effects. In our study, both diesel particles and particulate matter induced a diabetic phenotype, indicating that they share the chemical compound mediating the diabetic phenotype. However, DEP induced more pronounced effects than PM in terms of glucose tolerance, liver lipids or lung inflammation. This could potentially be due to a higher concentration of the hazardous substance(s) in DEP compared to PM. Polycyclic aromatic hydrocarbons (PAHs), for example, are present in both DEP (NIST 1650b) and PM (NIST 1649b), however at higher concentrations in the DEP samples. Intriguingly, urinary biomarkers of PAHs have also been associated with diabetes [42–44]. Besides, exposure to PAH has also been related to gut inflammation, similar to our findings [45].

Previous studies found that chronic DEP exposure affects lung tissue as shown by changes in immune cells or alveolar enlargement [46–49]. However, whether and how such changes in lung pathology are causally linked to the development of diabetes remains elusive. In our study, we found that particulate air pollution exposure via deep intratracheal instillations led to lung inflammation as shown by increased eosinophils and monocyte-derived macrophages. However, lung inflammation was not associated with the development of diabetes. We cannot exclude that the repeated intratracheal instillations led to subtle airway injuries that might have affected glucose homeostasis. However, intratracheal instillations were equally performed in control mice receiving PBS, which served as a reference point to estimate the effect of particulate air pollution exposure via the lung.

The association of air pollution exposure and different metabolic outcomes including diabetes has been widely established (Additional file 1: Table S1). Most studies so far used inhalation chambers, whereby lung and gut exposures occur simultaneously. Metabolic readouts comprise glucose intolerance, insulin resistance, increased liver lipids, dyslipidemia, and systemic inflammation. However, although these features are very similar between high-fat diet related and air pollution-induced diabetes, there is a big difference in terms of the temporal development of these changes. High-fat diet leads to glucose intolerance, insulin resistance, systemic and adipose tissue inflammation within only a few days [50], while changes in glucose tolerance upon air pollutants usually occur after long-term exposure, which suggests a different disease mechanism. Importantly, so far only few studies on air pollution-induced diabetes reported insulin levels and if so, mostly unstimulated insulin at a single timepoint (Additional file 1: Table S1). Also, for the interpretation of insulin, it is important to take into account a interdependency of glucose and insulin during the progression of type 2 diabetes [51]: Early in classical metabolic disease, insulin secretion is increased to ensure glucose uptake into peripheral tissues and glycogenesis. With time, beta-cells become exhausted and cannot compensate for the increased insulin demand, leading to hyperglycemia while insulin values revert to inadequately normal and only later reduced levels. We found that mice exposed to air pollution particles via gavage developed glucose intolerance and—despite elevated glucose levels—they were not able to mount a compensatory increase in insulin secretion, consistent with beta-cell dysfunction. To test mice in a “prestressed” condition, we used high fat diet (HFD) and a single injection of streptozocin (STZ) [34, 35]. We chose this model as it combines two key aspects of type 2 diabetes, namely insulin resistance (induced by HFD) and partial reduction of beta-cell mass (by STZ). As STZ partially destroys beta-cells, it limits beta-cell regeneration capacity, which could unmask an insulin secretion defect. Indeed, HFD/STZ mice exposed to particulate air pollutants via gavage developed glucose intolerance at an earlier timepoint compared to mice on standard diet, most likely due to an inability to compensate for the increased insulin demand. The finding of a beta-cell defect upon particulate air pollution exposure does not contradict the fact that air pollution is linked to type 2 diabetes as both beta-cell dysfunction and insulin resistance are key characteristics of type 2 diabetes. It rather spotlights the specific metabolic alteration induced by ingested air pollution particles. Similarly, another study using pre-treatment with PM_2.5_ followed by STZ treatment found a beta-cell defect as the predominant metabolic phenotype [52]. Potentially, this is one of the few studies reporting impaired insulin secretion as it became apparent due to the prior STZ treatment.

To our knowledge, hyperinsulinemic-euglycemic clamps, the gold standard to analyze insulin sensitivity, have not been performed in mice exposed to air pollutants. Instead, most studies used the HOMA-IR (Homeostatic Model Assessment for Insulin Resistance) as a measure for insulin resistance (Additional file 1: Table S1). Considering the interdependency of glucose and insulin during the progression of type 2 diabetes as mentioned above [51], the HOMA-IR could be inadequate to detect a slowly developing beta-cell secretory defects as its formula (“fasting insulin x fasting glucose/22.5 or 405”) could be increased solely by elevated glucose levels, while the insulin secretion defect would only be seen at a later time point. Therefore, hyperinsulinemic-euglycemic clamps will be required to assess whether (or to what extent) insulin resistance contributes to the diabetic phenotype instigated by air pollutants.

Interestingly, increased liver lipids developed upon both lung and gut exposure to DEP. This indicates that the pathogenesis of hepatic steatosis is independent of the exposure route and potentially mediated by other compounds and/or mechanisms as beta-cell dysfunction. On a molecular level, it was previously shown that air pollution-induced hepatic steatosis resulted from mitochondrial dysfunction with suppressed fatty acid oxidation, rather than increased de novo lipogenesis or fatty acid uptake [53]. Factors such as such the composition of the pollutants, genetic background, diet, or differences in gut microbiota of the mice could predispose susceptible disease models to develop liver steatosis upon air pollution exposure. While factors such as diet or gut microbiota could be susceptibility factors regarding the development of metabolic disease, they could also be altered by the air pollution particles themselves. Potentially, once accumulation of liver lipids and hepatic insulin resistance reach a certain threshold, systemic insulin resistance could occur upon air pollutants. Also, concomitant gut and lung exposures as in the case of inhalation chambers could have synergistic effects on metabolic outcomes and promote a more pronounced liver phenotype than what we observed by separate exposure routes.

One important question is the translatability of our mouse model to human disease. In terms of total particle deposition and clearance of particles in the lungs, mice and humans seem to be comparable, while there are some differences in the regional deposition and the clearance rate (higher in mouse compared to the human lung) [54]. In contrast, not much is known about potential differences in the mouse and human gastrointestinal tract in dealing with inhaled particles. Further studies are needed to address whether inhaled air pollution particles differentially affect human and mouse gut physiology.

## Conclusions

**Fig. 6:**
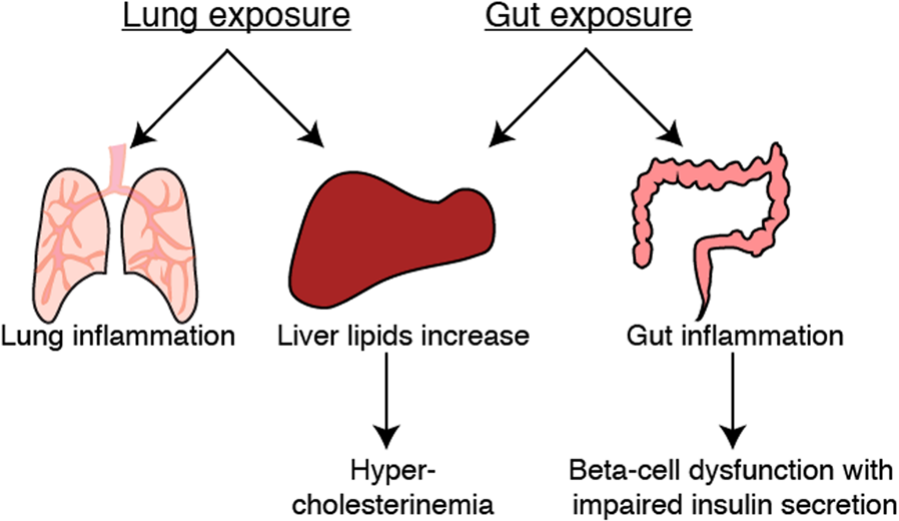
Different exposure route to diesel exhaust particles result in different outcomes. Lung exposure to air pollution particles leads to lung inflammation, whereas gut exposure induces gut inflammation and an insulin secretion defect. Both lung and gut exposures result in increased liver lipids with potential downstream effects on dyslipidemia

In sum, we show that air pollution-induced health effects are dependent on the exposure route (Fig. 6). Lung exposure caused lung inflammation, but did not result in the development of diabetes. Gut exposure to particulate air pollutants over a prolonged time period led to glucose intolerance due to a beta-cell secretory defect. Both lung and gut exposures were associated with increased liver lipids, potentially mediating hepatic insulin resistance in susceptible animal models. The gut—beta-cell crosstalk adds a new dimension to the pathophysiology of air pollution-induced diabetes and could potentially apply to other environmental factors related to diabetes development. Many questions remain open as for example the molecular mechanisms underpinning gut inflammation and beta-cell dysfunction upon exposure to pollutants. A link between an inflammatory response and beta-cell function is supported by previous studies showing that inflammatory cytokines induce beta-cell dedifferentiation [36] and a failure to coordinate insulin secretion within islets [55], potentially as an adaptive mechanism to escape beta-cell death under stress conditions [56]. Given the global burden of diabetes and the ever increasing plight of air pollution in numerous regions across the world, these findings are of high clinical significance towards building a healthier society.

## Methods

### Study design

We applied an approach that allowed us to separately expose the lung (by intratracheal instillation) and the gut (by gavage) to air pollution particles (see exposure protocol). As readout measures, we examined immune cells of different organs. All the experiments were carried out two to five times, except for lung exposure on standard diet and exendin (9–39), which were carried out once. Mice were grouped by weight matching, no further randomization was performed. Blinding was not feasible during the treatment phase, but results were analyzed in a blinded fashion whenever possible. Group and sample sizes for each experiment are indicated in the figure legends.

### Mice

Male C57BL/6N mice were obtained from Charles River Laboratories. Mice were maintained in specific pathogen-free conditions with free access to water and food. Standard diet was obtained by Granovit (Switzerland; Extrudat: 4.5% crude fat, 18.5% protein, 35% starch, 4.5% fibers, stored at room temperature). Exposures were started at 5–6 week of age.

To test mice in a “prestressed” condition, we used high-fat diet (HFD (58% coconut fat, 16.4% protein, 25.5% maltodextrin 10, Sucrose, 0.5% fibers, stored at − 20 °C), Cat# D12331, Research Diets) and a single intraperitoneal (i.p.) dose of streptozotocin (STZ; 120 mg/kg body weight, Cat# S0130 Sigma) after 4 weeks of HFD. The dose of STZ 120 mg/kg body weight is based on the literature [34, 35] and titrated in order to achieve hyperglycemia (glucose of approximately 15 mmol/L), but no insulin-dependency, cachexia or mortality (Fig. 2C, G for time course of body weight). We used this model as it combines two key aspects of type 2 diabetes, namely insulin resistance (induced by HFD) and partial reduction of beta-cell mass (by STZ). All animal procedures were approved by the local Animal Care and Use Committee and performed in accordance with Swiss Federal regulations.

### Exposure protocol

Diesel exhaust particles (DEP; NIST 1650b, pH 6.6–6.8) or particulate matter (PM; NIST 1649b) dissolved in sterile PBS (Cat# D8537, Sigma), or PBS alone as control, were administered either to the lung by intratracheal instillation or to the gut by gavage starting at 5–6 weeks of age until sacrifice (for a detailed characterization of the chemical composition see embedded link). Intratracheal instillation was performed as previously described [57]. Suspension characteristics of the dissolved particles see Additional file 1: Table S2.

For gavage, mice received daily 12 µg DEP or PM (or 60 µg; dose escalation experiment, see Additional file 1: Figure S1) suspended in 200 µL sterile PBS or PBS as control. In the “prestressed” condition, 20 µg DEP daily were used. The rationale for the slightly higher dose in the “prestressed” setting was that we hypothesized that the time frame to develop glucose intolerance would be shorter in a “prestressed” setting induced by HFD/STZ. We therefore assumed a shorter exposure period and chose a slightly higher weekly exposure dose to approximate the total deposition dose of the standard diet model. While gavage was performed 5 days a week, intratracheal instillation was conducted only twice weekly. To keep both models comparable, the same weekly dose of total 60 µg or 100 µg (in case of diabetic mice) DEP or PM, respectively were instilled. The lower dose represents an average daily dose of 8.6 µg/mouse and approximately equates a daily inhalation exposure of about 160 µg/m^3^ (calculated by the daily exposure divided by the daily inhaled air volume).

The calculation of the daily inhaled air volume was based on a minute volume-to-body weight ratio of 1.491 (L/(min*kg)):

$$1.491\frac{L}{min*kg}*0.025kg*60min*24h=53\frac{L}{day}=0.053\frac{{m}^{3}}{day}$$

### Glucose and insulin tolerance tests (GTTs/ITTs)

For GTTs, mice received a glucose bolus i.p. (2 g/kg body weight, Braun) after 6 h of fasting. Blood glucose was measured after 0, 15, 30, 60, 90 and 120 min using a freestyle lite glucometer (Cat#7091870, Abbott). Blood was collected at time points 0, 15 and 30 min for insulin measurements.

For ITTs, mice were fasted 3 h and injected with 1U/kg body weight insulin (Actrapid Penfill Insulin 100 IU/mL, Novo Nordisk). Glucose levels were measured at 0, 15, 30, 60, 90, and 120 min after injection.

For GLP-1 measurements, mice were i.p. injected with 25 mg/kg body weight Sitagliptin (Cat# sc-364620, Santa Cruz) 30 min prior to oral glucose administration and blood collected in diprotein A (Cat# I9759, Bachem). To block GLP-1 signaling, synthetic exendin (9–39) 25 nM/kg body weight (Cat# H-8740, Bachem) was injected i.p. 1 min prior to GTT.

### Isolation and flow cytometric assessment of immune cells

Cells of the lung, adipose tissue and liver were isolated by enzymatic digestion as follows:

*Lung:* Lungs were perfused with PBS from the heart, isolated and minced first with scissors, followed by the gentleMACS program m_lung_01-02, digested 30 min at 37 °C on an orbital shaker with 0.15 WünschU/mg Liberase (Cat# 5401020001, Roche) and 0.1 mg/mL DNase I, and homogenized by using gentleMACS program m_lung_02_01. Lung cells were enriched for leukocytes by Percoll gradient (40% /70%, 600 g, 20 min, minimal brake). Gating see Additional file 1: Figure S2A.

*Adipose tissue*: Epididymal adipose tissue was minced and digested with 1.5 mg/mL collagenase IV (Cat# LS004189, Worthington), 10 mM HEPES and 8.25 µg/mL DNase I for 20–25 min on a thermomixer with 400 rpm. Erythrocytes were removed by red cell lysis buffer (154 mM NH4Cl, 10 mM KHCO3, 0.1 mM EDTA). Adipose tissue macrophages were gated as CD45^+^F4/80^+^CD11b^+^ and subdivided into subpopulations double negative (DN), pro-inflammatory M1a (CD11c^+^CD206^−^) and M1b (CD11c^+^CD206^int^), and anti-inflammatory M2 (CD11c^− to low^CD206^+^). Gating see Additional file 1: Figure S2B.

*Liver macrophages*: One liver lobe was minced and digested using 1.5 mg/mL collagenase IV, 10 mM HEPES and 8.25 µg/mL DNase I, on a thermomixer with 400 rpm. After 15 min, the tissue was mechanistically dissected by pipetting up and down with a 1 mL pipette, and digested for another 15 min, followed by filtration and Percoll gradient. Gating see Additional file 1: Figure S2C.

Cell analysis was performed on a FACS LSRII Fortessa (BD Biosciences). Acquired data were analyzed using FlowJo software (Version 9.9 or higher), TreeStar Inc. Ashland, OR, USA).

From Biolegend, we obtained antibodies against CD11b (M1/70), CD11c (N418), MHCII (M5/114.14.2), Ly6C (HK1.4), CD45 (30-F11), F4/80 (BM8), CD103 (2E7), CD24 (M1/69), CD64 (X54-5/7.1), CD3 (145-2C11), CD19 (6D5), NK1.1 (PL136), Ly6G (1A8) and CD206 (C068C2). mAb for CCR2 (475,301) was purchased from R&D. mAb for Siglec F (E50-2440) was obtained from BD. For further details see Additional file 1: Table S3.

### Primary colon crypt cultures

Colon tissue was digested (0.3 mg/mL collagenase XI (Cat# C7657, Sigma)) and collected as previously described [58] and cells uniformly distributed on a 24-well plate coated with 0.1% gelatin (Sigma). To assess GLP-1 release, cells were pre-incubated with Krebs Ringer solution (0.1 mM glucose) 37 °C, 15 min, followed by 2 h of collection in low (0.1 mM) or high (11.1 mM) glucose, with concomitant DEP (125 µg/mL) or PBS treatment. GLP-1 secretion was normalized to protein content (Pierce BCA protein assay kit, Cat# 23,227, Thermo Fischer Scientific).

### Gene expression analysis

RNA isolation was performed using the NucleoSpin RNA (Cat# 740955, Macherey Nagel) or the RNeasy Plus Universal Mini kit (Cat# 73404, QIAGEN). Reverse transcription was performed with GoScript™ (Cat# A5003, Promega). GoTaq qPCR Master Mix (Cat# A4472919, Promega) was used for real-time PCR (ViiA7, Thermo Fisher Scientific). Primer sequences (Microsynth) are listed in Additional file 1: Table S4.

### Protein expression analysis

Plasma insulin, active GLP-1, TNF and IL-6 were quantified by electrochemiluminescence (MESO SECTOR S 600) using kits from MesoScale Diagnostics (Cat# K152BZC, K150JWC and K15048, respectively).

### Liver enzymes and lipids

*Total liver lipids by sulfo-phospho-vanillin reaction* [59]: Liver tissue was homogenized in PBS, 2:1 Chloroform-MeOH was added and the samples spun 5 min at 4 °C, 3400 rpm. The bottom phase was collected, dried, cooled down on ice, after adding H2SO4 boiled at 90 °C, 10 min and cooled down on ice. After 40 min incubation with Vanillin-reagent, the samples were measured at 550 nm (BioTek instruments).

Liver enzymes and blood lipids were measured in plasma on the c502/c702 modules of the Cobas 8000 series (Roche Diagnostics).

### Glucose-stimulated insulin secretion (GSIS)

Mouse islets were isolated as previously described [60] by collagenase digestion (1.5 mg/mL Collagenase IV) and subsequently purified by filtration and hand-picking. Islets were cultured free-floating in RPMI-1640 medium containing 11 mmol/L glucose and 10% FCS overnight, washed, incubated 90 min in Krebs–Ringer buffer containing 2.8 mmol/L glucose and 0.5% BSA prior to incubation in Krebs–Ringer buffer containing 2.8 or 20.0 mmol/L glucose and 0.5% BSA for 1 h. Islet insulin content was extracted with 0.18 mol/L HCl in 70% ethanol to determine insulin content.

### Beta-cell mass

For heat-induced antigen retrieval, 5 µm thick sections were boiled for 30 min at 93 °C in 1 × epitope retrieval solution (Cat# AR9961, Biosystems). The slides were stained overnight at 4 °C with the primary antibody for insulin (Cat# A0564, Agilent), washed twice in PBS (5 min), stained for 2 h at room temperature with a CD45 antibody (Rat Anti-CD45; 30-F11, Cat# 553076, BD Bioscience) and washed twice with PBS (5 min). The secondary antibodies were applied for 2 h at room temperature (Alexa647 goat anti-guinea pig IgG and Alexa555 goat anti-rat IgG; Cat# A-21450 and A-21434, respectively, Thermo Fisher Scientific), washed twice with PBS, before mounting with a fluorescence mounting media (Cat# S3023, Dako). Pictures were acquired using a Nikon microscope at 4× magnification.

Images were analyzed using Fiji software (1.52n with Java 1.8.0_172). Analysis was performed in a semi-automated way; ilastik software (Version 1.3.2, https://www.ilastik.org/) was trained twice, once to recognize pancreas area excluding background and lymph nodes, and the second time to recognize islets. The masks ilastik generated were used to quantify the areas with Fiji (1.52n with Jaca 1.8.0_172). Beta-cell mass was defined as area of insulin positive cells/area of pancreas*weight of the pancreas. Two sections per animal were quantified.

### Quantification and statistical analysis

Data are expressed as mean ± SEM. Two-way ANOVA was used to determine statistical significances in GTT and insulin over time. Unpaired Mann–Whitney U test was used for statistical significance (GraphPad Prism, Version 8). p-value < 0.05 was considered statistically significant. GTT data show one representative experiment, all other data are pooled.

## Supplementary Information

**Additional file 1**. Figures and Tables.

## Data Availability

Data analyzed during the current study are available from the corresponding author upon request.

## Abbreviations

ALAT: Alanine transaminase
AP: Alkaline phosphatase
AUC: Area under the curve
BSA: Bovine Serum Albumin
DEP: Diesel exhaust particles
EDTA: Ethylenediaminetetraacetic acid
FACS: Fluorescence Activated Cell Sorting
GLP-1: Glucagon-like peptide-1
GTT: Glucose tolerance test
H&E: Hematoxylin and eosin
HOMA-IR: Homeostatic Model Assessment for Insulin Resistance
ITT: Insulin tolerance test
i.p.: Intraperitoneal
PBS: Phosphate-buffered saline
SEM: Standard Error of the Mean
STZ: Streptozotocin
