# Supplementary material for: Lung versus gut exposure to air pollution particles differentially affect metabolic health in mice
Source: Part Fibre Toxicol. 2023 Mar 9;20:7. doi: 10.1186/s12989-023-00518-w (PMC9996885; doi:10.1186/s12989-023-00518-w)
Supplement: Supplementary file 1 — Additional file 1. Figures and Tables. [file 12989_2023_518_MOESM1_ESM.docx]

**Additional file 1: Figures**

**Lung versus gut exposure to air pollution particles**

**differentially affect metabolic health in mice**

**Authors:** Angela J. T. Bosch^1^, Theresa V. Rohm^1^, Shefaa AlAsfoor^1^, Andy J. Y. Low^1^, Zora Baumann^1^, Neena Parayil^1^, Marc Stawiski^1^, Leila Rachid^1^, Thomas Dervos^1^, Sandra Mitrovic^2^, Daniel T. Meier^1^, Claudia Cavelti-Weder^1,3,4*^

**Affiliations:**

^1^Department of Biomedicine, University of Basel, 4031 Basel, Switzerland.

^2^Department of Laboratory Medicine, University Hospital Basel, 4031 Basel, Switzerland.

^3^Clinic of Endocrinology, Diabetes and Metabolism, University Hospital Basel, 4031 Basel, Switzerland.

^4^Department of Endocrinology, Diabetology and Clinical Nutrition, University Hospital Zurich (USZ) and University of Zurich (UZH), Zurich, Switzerland.

**
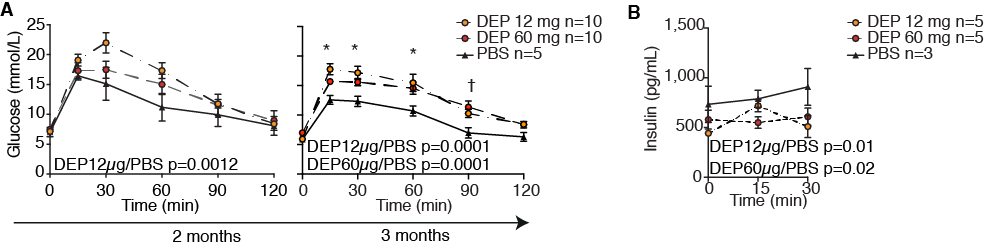
**

**Additional file 1:**  **Figure S1: Impaired glucose tolerance is independent of the DEP dose in mice fed a standard diet.** Wild-type mice fed a standard diet were treated with 12µg or 60µg diesel exhaust particles (DEP) or PBS 5 times per week via gavage for up to 6 months. **A** Glucose tolerance test (GTT) after 2 and 3 months of exposure. **B** Insulin after 3 months of exposure to 12µg or 60µg DEP or PBS. Data are presented as mean±SEM of 3-10 mice per group from one representative experiment. GTT and insulin values were compared by two-way ANOVA, *p<0.05. * indicates significances between 12 µg DEP and PBS controls, † indicates significances between 60µg DEP and PBS controls.

**
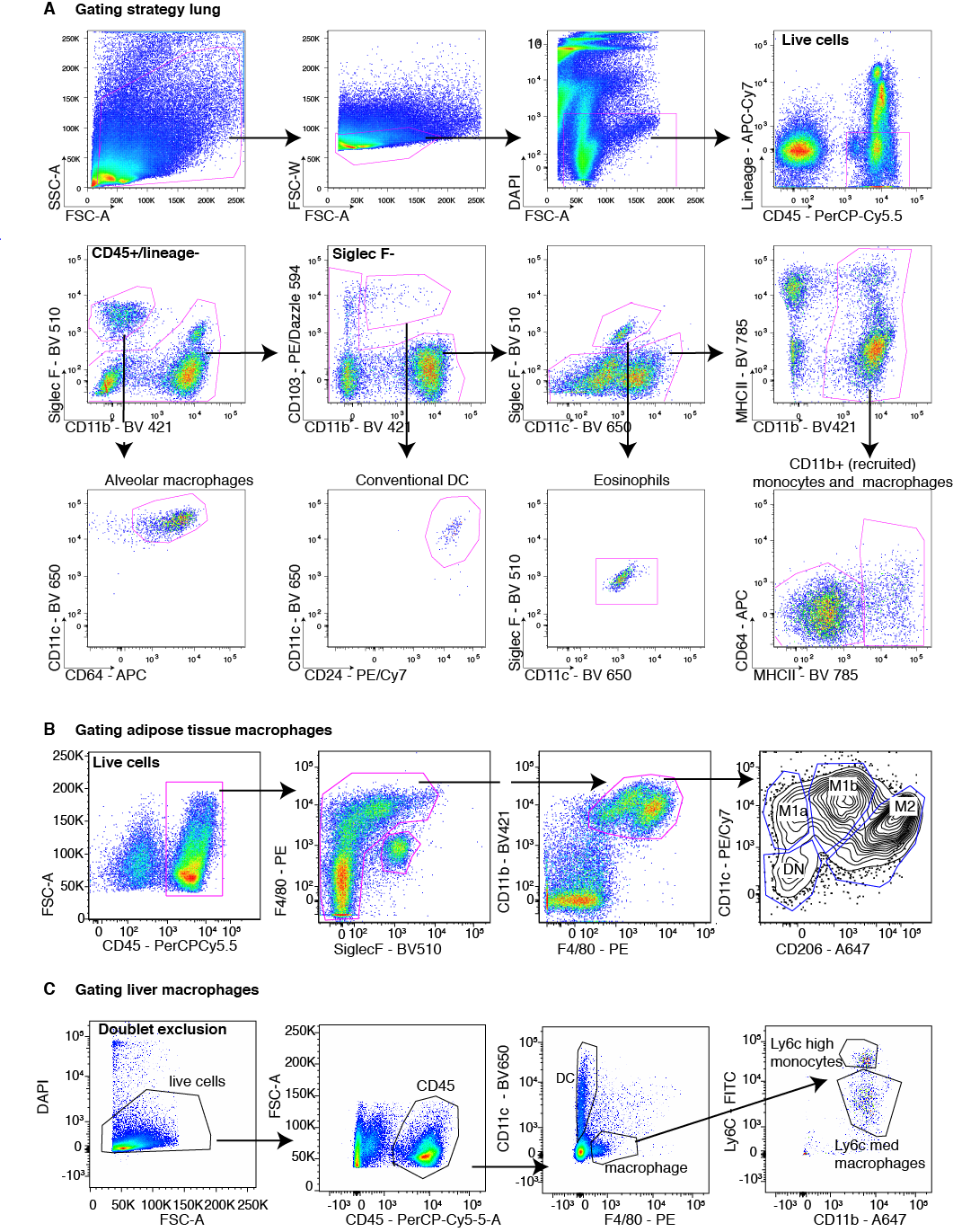
**

**Additional file 1: Figure** **S2: Gating strategy of immune cells in the lung, adipose tissue and liver.** All gating strategies included a gate for lymphocytes, followed by doublet exclusion and exclusion of dead cells (only shown in panel A). **A** Lung cells were gated for lymphocytes followed by doublet exclusion and exclusion of dead cells. Afterwards, the cells were gated for CD45^+^ and lineage^-^ cells. Alveolar macrophages were gated according to SiglecF und CD64 expression. Conventional DC were defined as CD11b^low^, and high for CD103, CD24 and CD11c. Eosinophils were defined as SiglecF^+^, CD11c^high^ and macrophages were defined as CD11b^high^ positive cells. **B** Adipose tissue cells were gated for the expression of CD45^+^ followed by the expression of F4/80 and SiglecF. Eosinophils were defined as SiglecF^+^ F4/80^low^. CD11b and F4/80 double positive cells were further analyzed for the expression of CD206 and CD11b. **C** Liver cells were gated for the expression of CD45^+^ followed by gating for F4/80. F4/80 positive cells were further divided into Ly6C+ monocytes and Ly6C intermediate macrophages.

**
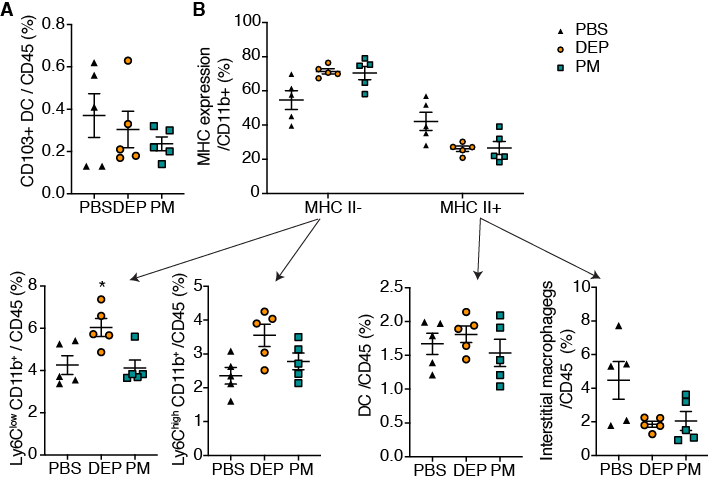
**

**Additional file 1: Figure S3: Lung inflammation upon air pollution exposure via intratracheal instillation in mice fed a standard diet.**  Wild-type mice were fed a standard diet and intratracheally instilled with 30µg diesel exhaust particles (DEP), particulate matter (PM) or PBS twice weekly for 6 months. **A** Frequency of CD103^+^ dendritic cells in lung of mice intratracheally treated with PBS, DEP or PM for 6 months. **B** CD11b^+^ cells of the lung, separated into MHC II^-^ and MHC II^+^ cells, and their corresponding subpopulations. MHC II^-^ CD11b^+^ cells were further divided into Ly6C^low^ or Ly6C^high^ expressing macrophages, while MHCII^+^CD11b^+^ cells were further distinguished into dendritic cells and interstitial macrophages. Data are presented as mean±SEM of 5 mice per group from one experiment compared by a two-tailed, unpaired Mann-Whitney U test (*p<0.05).


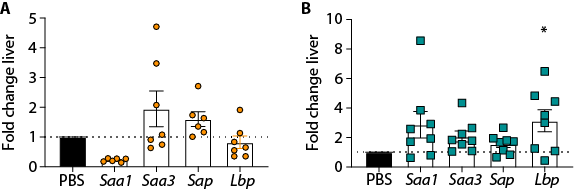


**Additional file 1: Figure S4: Acute phase genes in liver after oral exposure to air pollution in mice fed a standard diet.** Wild-type mice were fed a standard diet and treated with 12µg diesel exhaust particles (DEP), particulate matter (PM) or PBS 5 times per week via gavage for up to 9 months. Gene expression of acute phase genes in liver of DEP (**A**) or PM (**B**) exposed animals. Data of one experiment are presented as mean±SEM (n=5 PBS, n=7 DEP and n=8 PM) and compared by a two-tailed, unpaired Mann-Whitney U test (*p<0.05).

**Additional file 1: Tables**

| **Study** | **Exposure dose and time** | **Strain** | **Diet** | **Glucose tolerance** | **Insulin secretion** | **Insulin sensitivity** | **Hepatic insulin resistance** | **Liver lipids/ steatosis** | **Systemic lipids** | **Systemic inflammation** |
| --- | --- | --- | --- | --- | --- | --- | --- | --- | --- | --- |
| Tan et al (1) | Inhalation PM_2.5_  85 μg/m^3^  5x/wk 6 hr  6 wk | C57BL/6 | HFD & CD | *Not reported* | *Not reported* | *Not reported* | *Not reported* | **HFD: Increased inflammation**  CD: no change | *Not reported* | *Not reported* |
| Xu et al (2) | Inhalation PM_2.5_  111 μg/m^3^  5x/wk 6 hr  10 wk | C57BL/6J | HFD & CD | HFD: GTT not impaired  **CD: GTT impaired** | *Not reported* | HFD: HOMA-IR unchanged  **CD: HOMA-IR increased** | *Not reported* | *Not reported* | *Not reported* | **Plasma TNF increased**, IFN-γ, MCP-1, RANTES unchanged |
| Rajagopalan et al (3) | Inhalation PM_2.5_  60-120 μg/m^3^ 5x/wk 6 hr  14 wk | C57BL/6J | CD | **GTT impaired** | *Not reported* | **ITT impaired** | *Not reported* | *Not reported* | *Not reported* | *Not reported* |
| Sun et al (4) | Inhalation PM_2.5_  73μg/m^3^  5x/wk 6hr  24 wk | C57BL/6J | HFD | **GTT impaired** | **Fasting insulin increased** | **HOMA-IR increased** | *Not reported* | *Not reported* | TC & TG no change, HDL reduced | *Not reported* |
| Liu et al (5) | Inhalation PM_2.5_  103μg/m^3^  5x/wk 6 hr  8 wk | KKay | CD | **GTT impaired at 5 wks**, unchanged at 8 wks | *Not reported* | ITT unchanged at 5 wks, not conclusive at 8 wks | Liver p-Akt/Akt reduced at 5 wks, but not 8 wks | *Not reported* | *Not reported* | Plasma IL-12p70, IFN-γ, IL-6, TNF, MCP-1 unchanged |
| Liu et al (6) | Inhalation PM_2.5_  101μg/m^3^  5x/wk 6 hr  26 wk | C57BL/6J & CCR2-/- | CD | GTT not impaired | *Not reported* | **ITT impaired at 3 and 6 months** | **Liver p-Akt/Akt reduced** | *Not reported* | *Not reported* | **Plasma IFN-γ increased,** but IL-12p70, IL-6, TNF, MCP-1 unchanged |
| Xu et al (7) | Inhalation PM_2.5_  64 μg/m^3^  5x/wk 6 hr  12 wk | wt & Nrf2-/- C57BL/6 | CD | **GTT impaired** | *Not reported* | **HOMA-IR increased** | **Liver p-Irs1/Irs1 (Ser) increased**; p-Akt/Akt unchanged | *Not reported* | *Not reported* | *Not reported* |
| Xu et al (8) | Inhalation PM_2.5_  94 μg/m^3^  5x/wk 6 hr  10 months | C57BL/6J | CD | **GTT impaired** | Fasting insulin not changed | **HOMA-IR increased** | **Liver p-Akt/Akt reduced** | *Not reported* | *Not reported* | Plasma IL-12p70, IFN-γ, TNF, MCP-1 unchanged |
| Pan et al (9) | Inhalation PM_2.5_  324 μg/m^3^  7x/wk 10 hr  12 wk | C57BL/6  & db/db | CD | **GTT impaired** | *Not reported* | **ITT impaired and HOMA-IR increased** | *Not reported* | *Not reported* | **TC & TG increased, HDL reduced** | *Not reported* |
| Liu et al (10) | Inhalation PM_2.5_  117ug/m^3^  5x/wk 6hr  17 wk | C57BL/6J & CCR2-/- | HFD | **GTT impaired** | *Not reported* | **ITT impaired** | Liver p-Akt/Akt(Ser) and p-Irs1/Irs1 unchanged | **Liver TG increased** | **Plasma TG increased** | *Not reported* |
| Zheng et al (11) | Inhalation PM_2.5_  74.6 μg/m^3^  5x/wk 12 hr  3 & 10 wk | C57BL/6 | CD | **GTT impaired** at 10 wks (but not 3 wks) | **Fasting insulin increased** | **HOMA-IR increased** | **Liver p-Irs1/Irs1 (Ser) elevated, p-Akt/Akt reduced** | **Hepatic steatosis/ inflammation, liver TG/TC increased after 10 wks** | **TG increased,** HDL unchanged | **Plasma TNF increased** |
| Xu et al (12) | Inhalation PM_2.5_  115 or 230 μg/m^3^  5x/wk 6 hr  24 wk | C57BL/6 | CD | **oGTT impaired** | **Insulin increased** | **ITT impaired** | *Not reported* | **Hepatic steatosis/ inflammation/ oxidative stress,** | **TG/TC increased; HDL reduced** | **Different inflammation markers increased** |
| Ding et al (13) | Inhalation PM_2.5_  132 or 192 μg/m^3^  7x/wk 6 hr  5 months | C57BL/6J | HFD & CD | *Not reported* | *Not reported* | *Not reported* | *Not reported* | **Hepatic steatosis/ TG/TC increased** | TG/TC unchanged | Plasma TNF, IL-6 unchanged |
| Yin et al (14) | Inhalation DEP  unclear μg/m^3^  5x/wk 6 hr  16 wk | ApoE-/- | CD | *Not reported* | *Not reported* | *Not reported* | *Not reported* | **Liver TG increased,** TC unchanged | **TG/TC increased** | *Not reported* |
| Zhang et al (15) | Inhalation PM_2.5_ 103 μg/m^3^  12 wk | C57BL/6; multiple STZ injections | CD | **Fasting glucose increased** | **Insulin decreased** | *Not reported* | *Not reported* | **Liver TC increased** | **TC increased** | Plasma IL-1 TNF unchanged |
| Wang et al (16) | Oropharynx instillation PM_2.5_  1 mg/mL (20 μl) every other day, dose equivalent to 335 μg/m^3^/d | C57BL/6J (female) | CD | **oGTT impaired** | *Not reported* | ITT not impaired | **Liver p-Akt/Akt reduced** | *Not reported* | *Not reported* | *Not reported* |
| Chen et al (17) | Intratracheal instillation DEP 20 μg in 50 μl  3x/wk for 19 wk (160 μg/m^3^) | SFTPCrtTA +/− tetO-cre +/− (-/-) IKK2fl/fl | CD | **GTT impaired** | **Insulin levels increased**  **(0,15,30min)** | **ITT impaired** | **Liver p-Akt/Akt reduced**, **p-Irs1/Irs1 (Ser) increased** | *Not reported* | *Not reported* | **Plasma TNF and IL-6 increased,** IL-1β unchanged |

**Additional file 1:** **Table S1: Overview on metabolic outcomes upon exposure to air pollution in mice.** Ref: reference, DEP: diesel exhaust particles, PM: particulate matter, hr: hours, wk: weeks, CD: Control diet, HFD: high-fat diet, (o)GTT: (oral) glucose tolerance test, ITT insulin tolerance test, TC: total cholesterol, TG: triglycerides, HDL: high density lipoproteins

| **Concentration**  **(mg/ml)** | **Number of particles in 1µl** | **Diameter, SD (nm)** | **Number of agglomerates** | **Diameter, SD, range (nm)** |
| --- | --- | --- | --- | --- |
| Series 1 | | | | |
| **1** | 12 | 30.5, 5.7 | 7 | 197, 59, 113 - 268 |
| **0.6** | 10 | 34.7, 7.5 | 8 | 358, 210. 169 - 795 |
| **0.3** | 10 | 34.7, 5.5 | 5 | 358, 181, 183 - 602 |
| **0.06** | 14 | 35.0, 9.0 | 6 | 180, 95, 98 – 366 |
| **TOTAL** | **45** | **33.7** | **26** |  |
|  |  |  |  |  |
| Series 2 | | | | |
| **1** | 22 | 34.3, 8.7 | 10 | 274, 90, 119 - 406 |
| **0.6** | 24 | 40.1, 11.1 | 4 | 303, 145, 175 - 441 |
| **0.3** | 15 | 46.3, 12.1 | 2 | 237, 123, 150 - 324 |
| **0.06** | 6 | 49.0, 13.3 | 7 | 181, 80, 85 - 325 |
| **Total** | **67** | **42.4** | **23** |  |

Additional file 1: Table S2: Suspension characteristics of dissolved particles. Diesel particles form aggregates in solution independent of the concentration tested.

| REAGENT or RESOURCE | SOURCE | IDENTIFIER |
| --- | --- | --- |
| Antibodies | | |
| Anti-mouse CD16/32 (93); Dilution (1:100) | Biolegend | Cat#101321; RRID: [AB_2103871](http://antibodyregistry.org/AB_2103871) |
| Anti-mouse CD11c (N418) BV650; Dilution (1:100) | Biolegend | Cat#117339; RRID: [AB_2562414](http://antibodyregistry.org/AB_2562414) |
| Anti-mouse CD11c (N418) PE-Cy7; Dilution (1:120) | Biolegend | Cat#117318; RRID: [AB_493568](http://antibodyregistry.org/AB_493568) |
| Anti-mouse CD11b (M1/70) BV421; Dilution (1:40) | Biolegend | Cat#101236; RRID: [AB_11203704](http://antibodyregistry.org/AB_11203704) |
| Anti-mouse CD45 (30-F11) PerCP-Cy5.5; Dilution (1:300) | Biolegend | Cat#103131; RRID: [AB_893344](http://antibodyregistry.org/AB_893344) |
| Anti-mouse I-A/I-E (M5/114.15.2) BV785; Dilution (1:500) | Biolegend | Cat#107645;  RRID: [AB_2565977](http://antibodyregistry.org/AB_2565977) |
| Anti-mouse Ly6C (HK1.4) FITC; Dilution (1:350) | Biolegend | Cat#128005;  RRID: [AB_1186134](http://antibodyregistry.org/AB_1186134) |
| Anti-mouse CCR2 (475301) PE; Dilution (1:25) | R&D Systems | Cat#FAB5538P;  RRID: [AB_10718414](http://antibodyregistry.org/AB_10718414) |
| Anti-mouse CD103 (2E7) PE-Dazzle594; Dilution (1:80) | Biolegend | Cat#121430;  RRID: [AB_2566493](http://antibodyregistry.org/AB_2566493) |
| Anti-mouse CD24 (M1/69) PE-Cy7; Dilution (1:160) | Biolegend | Cat#101821;  RRID: [AB_756047](http://antibodyregistry.org/AB_756047) |
| Anti-mouse CD64 (X54-5/7.1) APC; Dilution (1:60) | Biolegend | Cat#139306;  RRID: [AB_11219391](http://antibodyregistry.org/AB_11219391) |
| Anti-mouse CD3 (145-2C11) APC-Cy7; Dilution (1:35) | Biolegend | Cat#100330;  RRID: [AB_1877170](http://antibodyregistry.org/AB_1877170); |
| Anti-mouse Nk1.1 (PK136) APC-Cy7; Dilution (1:40) | Biolegend | Cat#108723;  RRID: [AB_830870](http://antibodyregistry.org/AB_830870) |
| Anti-mouse CD19 (6D5) APC-Cy7; Dilution (1:600) | Biolegend | Cat#115530; RRID: [AB_830707](http://antibodyregistry.org/AB_830707) |
| Anti-mouse Siglec F (E50-2440) BV510; Dilution (1:60) | BD Biosciences | Cat#740158;  RRID: [AB_2739911](http://antibodyregistry.org/AB_2739911) |
| Anti-mouse F4/80 (BM8) PE; Dilution (1:100) | Biolegend | Cat#123110;  RRID: [AB_893486](http://antibodyregistry.org/AB_893486) |
| Anti-mouse CD206 (C068C2) A647; Dilution (1:160) | Biolegend | Cat#141712;  RRID: [AB_10900420](http://antibodyregistry.org/AB_10900420) |
| Guinea pig-anti-insulin; Dilution (1:100) | Agilent | Cat#A0564; [AB_10013624](https://antibodyregistry.org/search.php?q=AB_10013624) |
| Rat Anti-CD45 (30-F11) Monoclonal Antibody, Unconjugated; Dilution (1:100) | BD Biosciences | Cat#553076;  [RRID:AB_394606](https://antibodyregistry.org/search.php?q=AB_394606) |
| Goat anti-Guinea Pig IgG (H+L) Highly Cross-Adsorbed Secondary Antibody, Alexa Fluor 647; Dilution (1:100) | Thermo Fisher Scientific | Cat#A-21450; [RRID:AB_2735091](https://antibodyregistry.org/search.php?q=AB_2735091) |
| Goat anti-Rat IgG (H+L) Cross-Adsorbed Secondary Antibody, Alexa Fluor 555; Dilution (1:100) | Thermo Fisher Scientific | Cat#A-21434; [RRID:AB_2535855](https://antibodyregistry.org/search.php?q=AB_2535855) |
| **Chemicals, Peptides, and Recombinant Proteins** | | |
| Streptozocin (STZ) | Sigma | Cat# S0130 |
| 60% coconut-based HFD | Research Diets | Cat#D12331 |
| Diesel exhaust particles (DEP) | National Institute of Standards and Technologies/Sigma | Cat# NIST1650B |
| Patriculate matter (PM) | National Institute of Standards and Technologies/Sigma | Cat# NIST1649B |
| Diprotein A | Sigma- Aldrich | Cat#I9759, CAS:90614-48-5 |
| Sitagliptin phosphate monohydrate | Santa Cruz Biotechnology | Cat#sc-364620, CAS: 654671-77-9 |
| Exendin (9-39) | Bachem | Cat#H-8740, CAS: 133514-43-9 |
| Collagenase IV | Worthington | Cat# LS004189 |
| Collagenase XI | Sigma-Aldrich | Cat# C7657 |
| Liberase | Roche | Cat# 5401020001 |
| DNase 1 | Roche | Cat# 11284932001 |
| Percoll | GE Healthcare | Cat# GE17-0891-01 |
| **Critical Commercial Assays** | | |
| Mouse/rat insulin kit | MesoScale Diagnostics | Cat#K152BZC |
| active GLP-1 (ver. 2) kit | MesoScale Diagnostics | Cat#K150JWC |
| V-Plex custom mouse cytokine proinflammatory panel 1 mouse TNF-α and mouse IL-6 | MesoScale Diagnostics | Cat#K15048 |
| NucleoSpin RNA kit | Macherey Nagel | Cat# 740955 |
| RNeasy Plus Universal Mini kit | Qiagen | Cat# 73404 |
| GoScript^TM^ | Promega | Cat# A5003 |
| GoTaq qPCR Master Mix | Promega | Cat# A4472919 |
| Pierce BCA protein assay kit | Thermo Fisher Scientific | Cat# 23227 |
| **Deposited Data** | | |
| scRNA-seq Data | This paper | GSE133406 |
| **Experimental Models: Organisms/Strains** | | |
| Mouse, C57BL/6NCrl | Charles River laboratories | RRID:IMSR_CRL:027 |
| **Oligonucleotides** | | |
| Primers for qPCR -> Suppl. Table 1 | Microsynth | N/A |
| **Software and Algorithms** | | |
| Fiji software ImageJ 1.52n with Java 1.8.0_172 |  | <https://imagej.net/Fiji> |
| Ilastik (version 1.3.2) |  | [www.ilastik.org](http://www.ilastik.org) |
| Flow jo (version 9.9 or higher) | Becton Dickinson & Company (BD) | https://flowjo.com |
| BD FACS Diva (version 8.0.1) | Becton Dickinson & Company (BD) | <https://www.bdbiosciences.com/en-us/instruments/research-instruments/research-software/flow-cytometry-acquisition/facsdiva-software> |
| Prism 8 | GraphPad Software, LLc. | https://www.graphpad.com |
| R version 3.6 | The R Foundation | <https://www.r-project.org> |

Additional file 1: Table S3: Reagent and resource table.

| **Gene** | **Forward Primer** | **Reverse Primer** |
| --- | --- | --- |
| **Housekeeping genes** | | |
| ***B2m*** | 5′ TTCTGGTGCTTGTCTCACTGA | 5′ CAGTATGTTCGGCTTCCCATTC |
| ***Ppia*** | 5′ GAGCTGTTTGCAGACAAAGTTC | 5′ CCCTGGCACATGAATCCTGG |
| **Inflammation markers** | | |
| ***Tnf*** | 5′ ACTGAACTTCGGGGTGATCG | 5′ TGAGGGTCTGGGCCATAGAA |
| ***Il6*** | 5′ GGATACCACTCCCAACAGACCT | 5′ GCCATTGCACAACTCTTTTCTC |
| ***Il1b*** | 5′ GCAACTGTTCCTGAACTCAACT | 5′ ATCTTTTGGGGTCCGTCAACT |
| ***Cxcl1 (KC)*** | 5′ CTGGGATTCACCTCAAGAACATC | 5′ CAGGGTCAAGGCAAGCCTC |
| ***Il10*** | 5′ AGGCGCTGTCATCGATTTCTC | 5′ GCCTTGTAGACACCTTGGTCTT |
| ***Il18*** | 5′TCTTGCGTCAACTTCAAGGA | 5′GTGAAGTCGGCCAAAGTTGT |
| ***Il22*** | 5′TTG AGG TGT CCA ACT TCC AGC A | 5′AGC CGG ACG TCT GTG TTG TTA |
| ***Tgfb1*** | 5′CTCTCCACCTGCAAGACCAT | 5′CGAGCCTTAGTTTGGACAGG |
| ***Tgfb2*** | 5′GAAATACGCCCAAGATCGAA | 5′TGTCACCGTGATTTTCGTGT |
| ***Ifng*** | 5′GTCTCTTCTTGGATATCTGGAGGAACT | 5′GTAGTAATCAGGTGTGATTCAATGACGC |
| ***Il17*** | 5′ATC AGG ACG CGC AAA CAT GA | 5′TTG GAC ACG CTG AGC TTT GA |
| **Immune cells** | | |
| ***Cd68*** | 5′ GCAGCACAGTGGACATTCAT | 5′ AGAGAAACATGGCCC GAAGT |
| ***Adgre1 (Emr1)*** | 5′ GCC CAG GAGTGGAATGTCAA | 5′ CAGACACTCATCAACATCTGCG |
| ***Ly6c1*** | 5’GCA GTG CTA CGA GTG CTA TGG | 5’ACT GAC GGG TCT TTA GTT TCC TT |
| **Beta-cell identity** | | |
| ***Pdx1*** | 5′CCC CAG TTT ACA AGC TCG CT | 5′CTC GGT TCC ATT CGG GAA AGG |
| ***Foxo1*** | 5′GTA CGC CGA CCT CAT CAC CA | 5′TGC TGT CGC CCT TAT CCT TG |
| ***Ins2*** | 5′CCC TGC TGG CCC TGC TCT T | 5′AGG TCT GAA GGT CAC CTG CT |
| **Acute phase proteins** | | |
| ***Saa3*** | 5'GCC TGG GCT GCT AAA GTC AT | 5' TGC TCC ATG TCC CGT GAA C |
| ***Saa1*** | 5'GAGGACATGAGGACACCATTGC | 5'CCAGAGAGCATCTTCAGTGTTCC |
| ***SAP*** | 5'ATGCAGTGACTGTGTACCACG | 5'AGGGACACTCTCGCTGTCT |
| ***Lbp*** | 5'GATCACCGACAAGGGCCTG | 5'GGCTATGAAACTCGTACTGCC |

**Additional file 1:**  **Table S4: Primers sequences used for quantitative real time-PCR.**

**References**

1. Tan HH, Fiel MI, Sun Q, Guo J, Gordon RE, Chen LC, et al. Kupffer cell activation by ambient air particulate matter exposure may exacerbate non-alcoholic fatty liver disease. J Immunotoxicol. 2009;6(4):266-75.

2. Xu X, Yavar Z, Verdin M, Ying Z, Mihai G, Kampfrath T, et al. Effect of early particulate air pollution exposure on obesity in mice: role of p47phox. Arterioscler Thromb Vasc Biol. 2010;30(12):2518-27.

3. Rajagopalan S, Park B, Palanivel R, Vinayachandran V, Deiuliis JA, Gangwar RS, et al. Metabolic effects of air pollution exposure and reversibility. J Clin Invest. 2020;130(11):6034-40.

4. Sun Q, Yue P, Deiuliis JA, Lumeng CN, Kampfrath T, Mikolaj MB, et al. Ambient air pollution exaggerates adipose inflammation and insulin resistance in a mouse model of diet-induced obesity. Circulation. 2009;119(4):538-46.

5. Liu C, Bai Y, Xu X, Sun L, Wang A, Wang TY, et al. Exaggerated effects of particulate matter air pollution in genetic type II diabetes mellitus. Part Fibre Toxicol. 2014;11:27.

6. Liu C, Xu X, Bai Y, Zhong J, Wang A, Sun L, et al. Particulate Air pollution mediated effects on insulin resistance in mice are independent of CCR2. Part Fibre Toxicol. 2017;14(1):6.

7. Xu J, Zhang W, Lu Z, Zhang F, Ding W. Airborne PM2.5-Induced Hepatic Insulin Resistance by Nrf2/JNK-Mediated Signaling Pathway. Int J Environ Res Public Health. 2017;14(7).

8. Xu X, Liu C, Xu Z, Tzan K, Zhong M, Wang A, et al. Long-term exposure to ambient fine particulate pollution induces insulin resistance and mitochondrial alteration in adipose tissue. Toxicol Sci. 2011;124(1):88-98.

9. Pan K, Jiang S, Du X, Zeng X, Zhang J, Song L, et al. AMPK activation attenuates inflammatory response to reduce ambient PM2.5-induced metabolic disorders in healthy and diabetic mice. Ecotoxicol Environ Saf. 2019;179:290-300.

10. Liu C, Xu X, Bai Y, Wang TY, Rao X, Wang A, et al. Air pollution-mediated susceptibility to inflammation and insulin resistance: influence of CCR2 pathways in mice. Environ Health Perspect. 2014;122(1):17-26.

11. Zheng Z, Xu X, Zhang X, Wang A, Zhang C, Huttemann M, et al. Exposure to ambient particulate matter induces a NASH-like phenotype and impairs hepatic glucose metabolism in an animal model. J Hepatol. 2013;58(1):148-54.

12. Xu MX, Ge CX, Qin YT, Gu TT, Lou DS, Li Q, et al. Prolonged PM2.5 exposure elevates risk of oxidative stress-driven nonalcoholic fatty liver disease by triggering increase of dyslipidemia. Free Radic Biol Med. 2019;130:542-56.

13. Ding S, Yuan C, Si B, Wang M, Da S, Bai L, et al. Combined effects of ambient particulate matter exposure and a high-fat diet on oxidative stress and steatohepatitis in mice. PLoS One. 2019;14(3):e0214680.

14. Yin F, Gupta R, Vergnes L, Driscoll WS, Ricks J, Ramanathan G, et al. Diesel Exhaust Induces Mitochondrial Dysfunction, Hyperlipidemia, and Liver Steatosis. Arterioscler Thromb Vasc Biol. 2019;39(9):1776-86.

15. Zhang B, Yin R, Lang J, Yang L, Zhao D, Ma Y. PM2.5 promotes beta cell damage by increasing inflammatory factors in mice with streptozotocin. Exp Ther Med. 2021;22(2):832.

16. Wang N, Ma Y, Liu Z, Liu L, Yang K, Wei Y, et al. Hydroxytyrosol prevents PM2.5-induced adiposity and insulin resistance by restraining oxidative stress related NF-kappaB pathway and modulation of gut microbiota in a murine model. Free Radic Biol Med. 2019;141:393-407.

17. Chen S, Chen M, Wei W, Qiu L, Zhang L, Cao Q, et al. Glucose Homeostasis following Diesel Exhaust Particulate Matter Exposure in a Lung Epithelial Cell-Specific IKK2-Deficient Mouse Model. Environ Health Perspect. 2019;127(5):57009.
